# *Schistosoma haematobium* and soil-transmitted Helminths in Tana Delta District of Kenya: infection and morbidity patterns in primary schoolchildren from two isolated villages

**DOI:** 10.1186/s12879-016-1387-4

**Published:** 2016-02-03

**Authors:** Kariuki H. Njaanake, Birgitte J. Vennervald, Paul E. Simonsen, Henry Madsen, Dunstan A. Mukoko, Gachuhi Kimani, Walter G. Jaoko, Benson B. Estambale

**Affiliations:** 1 Department of Medical Microbiology, College of Health Sciences, University of Nairobi, P.O. Box 19676-00202, Nairobi, Kenya; 2 Department of Veterinary Disease Biology, Faculty of Health and Medical Sciences, University of Copenhagen, Dyrlægevej 100, 1870 Frederiksberg C, Denmark; 3 Division of Vector Borne & Neglected Tropical Diseases, Ministry of Public Health & Sanitation, P.O. Box 54840-00202, Nairobi, Kenya; 4 Centre for Biotechnology Research & Development, Kenya Medical Research Institute, P. O. Box 54840-00200, Nairobi, Kenya; 5 Jaramogi Oginga Odinga University of Science and Technology, P. O. Box 210-40601, Bondo, Kenya

**Keywords:** *Schistosoma haematobium*, Soil-transmitted helminths, Infections, Morbidity patterns

## Abstract

**Background:**

Schistosomes and soil-transmitted helminths (STH) (hookworm, *Trichuris trichiura* and *Ascaris lumbricoides*) are widely distributed in developing countries where they infect over 230 million and 1.5 billion people, respectively. The parasites are frequently co-endemic and many individuals are co-infected with two or more of the species, but information on how the parasites interact in co-infected individuals is scarce. The present study assessed *Schistosoma haematobium* and STH infection and morbidity patterns among school children in a hyper-endemic focus in the Tana River delta of coastal Kenya.

**Methods:**

Two hundred and sixty-two children aged 5–12 years from two primary schools were enrolled in the study. For each child, urine was examined for *S. haematobium* eggs and haematuria, stool was examined for STH eggs, peripheral blood was examined for eosinophilia and haemoglobin level, the urinary tract was ultrasound-examined for *S. haematobium*-related pathology, and the height and weight was measured and used to calculate the body mass index (BMI).

**Results:**

Prevalences of *S. haematobium*, hookworm, *T. trichiura* and *A. lumbricoides* infection were 94, 81, 88 and 46 %, respectively. There was no significant association between *S. haematobium* and STH infection but intensity of hookworm infection significantly increased with that of *T. trichiura*. Lower BMI scores were associated with high intensity of *S. haematobium* (difference =−0.48, *p* > 0.05) and *A. lumbricoides* (difference =−0.67, *p* < 0.05). Haematuria (both macro and micro) was common and associated with *S. haematobium* infection, while anaemia was associated with high intensity of *S. haematobium* (OR = 2.08, *p* < 0.05) and high hookworm infections OR = 4.75; *p* < 0.001). The majority of children had eosinophilia, which was significantly associated with high intensity of hookworm infection (OR = 5.34, *p* < 0.05). Overall 38 % of the children had ultrasound-detectable urinary tract morbidity, which was associated with high intensity of *S. haematobium* infection (OR = 3.13, *p* < 0.05).

**Conclusion:**

Prevalences of *S. haematobium* and STH infections among the primary school children were high and the parasites were responsible for significant morbidity. A clear synergistic interaction was observed between hookworm and *T. trichiura* infections. Increased coverage in administration of praziquantel and albendazole in the area is recommended to control morbidity due to these infections.

## Background

Schistosomiasis and soil-transmitted helminthiases are important neglected tropical diseases. It is estimated that schistosomes infect over 230 million people worldwide whereas soil-transmitted helminths (STH) such as hookworms, *Trichuris trichiura* and *Ascaris lumbricoides* infect over 1.5 billion resulting in 1.7 million and 5.2 million disability-adjusted life years, respectively [1–3]. Sub-Saharan Africa bears the largest burden of these infections, with primary school aged children from resource-poor communities being the most highly affected [3, 4].

Due to geographical overlap of transmission foci many individuals are co-infected with *Schistosoma* spp. and one or more of the STH species [5, 6]. However, marked heterogeneity in pattern of prevalence and co-infection exist [7–9]. Socioeconomic status, human behaviour, environmental factors and demography may be partly responsible for this [10]. Parasite-parasite and parasite-host interactions may also affect infection patterns in ways that are not yet clearly understood [11–14]. Increasing evidence moreover shows that such interactions may lead to not only increased worm burdens but increased morbidity as well [15].

Kenya is endemic for *S. haematobium*, *S. mansoni* and STH with foci of transmission scattered all over the country [9]. Existing data indicate that there is considerable intra-country spatial heterogeneity in prevalence of these infections with the coastal strip being one of the largest foci for *S. haematobium* and STH transmission in the country [7, 9]. Several studies on *S. haematobium* have been carried out in the southern and middle parts of the coastal strip, spanning from Kwale county in the south to Malindi county in the north [16–21]. Similarly, STH infections have been studied elsewhere in the coastal region [22–24]. There are, however, no comprehensive and systematic studies on schistosomiasis and STH infections in the northern part of coastal Kenya, which comprise an important endemic focus [9].

We conducted a study to document the pattern of *S. haematobium* and STH infections and resultant morbidity among schoolchildren in two isolated villages of Tana Delta District, coastal Kenya.

## Methods

### Study area

We chose two primary schools located in two isolated villages from where there had been no previous studies or treatment campaigns for schistosomiasis and STH: Kau in Kipini Division (2° 29’ 20” S, 40° 27’ 7” E) and Ozi in Garsen South Division (2° 31’ 9” S, 40° 27’ 40” E) in Tana Delta District, on the north coast of Kenya. Both villages are located on the banks of River Tana, about 5 km apart. The area is prone to seasonal flooding and, during the dry season, numerous swamps are scattered all over the area. The residents practice rice farming in the flood plains in the delta of River Tana as well as fishing in the river and swamps. Within each village, there is one primary school and children from each village attend their respective school. School enrolment in the two villages was high (about 90 %) but absenteeism was also high.

### Study design

This was a cross-sectional study of prevalence, intensity and morbidity due to *S. haematobium* and STH infections among schoolchildren aged between 5 and 12 years in the two villages. Three urine and stool samples were collected from each study participant (each urine and stool sample being collected on a different day), and examined for *S. haematobium* and STH eggs, respectively. One 2 ml venous blood sample was collected using a syringe and gauge 23 needle. Part of the blood (20 μl) was used to prepare a thin blood smear for malaria parasite examination and differential white blood cell count. Another 10 μl was used for haemoglobin level estimation. The rest was used for serum preparation for another study [25]. Weight and height of each child was recorded. Finally, their urinary tract was examined for *S. haematobium*-related morbidity using ultrasound. Prior to the start of the study, ethical approval was obtained from the University of Nairobi – Kenyatta National Hospital Ethics and Research Committee in Kenya (Approval No. P91/3/2009). Written informed consent for participation was obtained from each child’s parent or guardian. After the field sample collection, all children were treated with praziquantel (40 mg/kg) and albendazole (400 mg) in accordance with the national guidelines. Those who had not cleared the infections after the initial treatment were re-treated appropriately. Those presenting with other minor clinical manifestations including anemia were offered free treatment from the study team and those with clinical manifestations beyond the ability of the team were referred to the local health centre for medical attention.

### Urine examination for *S. haematobium* eggs

Ten millilitre of each of three consecutive urine samples from each child was filtered through a polycarbonate filter (12 μm pore-size; GE Water & Process Technologies Inc., USA) and the filter was examined microscopically for *S. haematobium* eggs [26]. The results, based on arithmetic mean of the three egg counts from each child, were classified into negative, light (1–49 eggs/10 ml urine) or heavy infections (≥50 eggs/10 ml urine) according to WHO [27, 28].

### Stool examination for soil transmitted helminth eggs

From each of the three stool samples obtained from each child, 41.7 mg was used to prepare a slide using standard Kato-Katz technique and was examined microscopically for the presence of hookworm, *T. trichiura* and *A. lumbricoides* eggs within 1 h of preparation [29]. Individual infections with STH were classified into three groups based on a mean of the three egg counts according to WHO classification [27, 28]. Infections with hookworm were classified as negative, light (1–1,999 eggs/g stool) and moderate to heavy (≥2,000 eggs/g stool). Infections with *T. trichiura* were classified as negative, light (1–999 eggs/g stool) and moderate to heavy (≥1,000 eggs/g stool) whereas those of *A. lumbricoides* were classified as negative, light (1–4,999 eggs/g stool) and moderate to heavy (≥5,000 eggs/g stool).

### Urine examination for haematuria

At the time of recruitment, participants were asked whether they had seen blood in their urine and during urine sample collection, each sample was examined visually for the presence of blood (macro-haematuria). The samples were also tested for presence or absence of occult blood (micro-haematuria) with dipstix (URISCAN®, YD Diagnostics, Korea) according to the manufacturer’s instructions. Change in colour of the test pad was noted within 1 min and interpreted according to a chart provided by the manufacturer.

### Haemoglobin

About 10 μl of venous blood was used to estimate haemoglobin concentration using a portable haemoglobinometer, which gives readings in g/dl, according to manufacturer’s instructions (HemoCue Hb 301, HemoCue®, Sweden). A new standard cuvette supplied together with the haemoglobinometer was used every day for quality control. Haemoglobin concentrations were expressed in g/dl and the results were recorded and categorised into two: normal (≥11.5 g/dl of blood) or anaemia (<11.5 g/dl of blood) [30].

### Blood cell count

A thin smear blood film was prepared using a small amount of each venous blood sample from each child and stained with Giemsa [26]. Differential cell count was performed on 100 white blood cells in each film and the numbers of each type of white blood cells expressed as a percentage of the total 100 white blood cells. Eosinophilia was defined as eosinophils above 7 % of white blood cells on the blood film (http://emedicine.medscape.com/article/2085133-overview#a1; Accessed 2nd June 2015).

### Anthropometry

The weight of each participant was measured to the nearest 0.5 kg using an electronic scale (Salter Electronic®), with the participant wearing only light clothes and no shoes. Height was measured to the nearest 1 cm using a portable stadiometer with the participants not wearing shoes. Body mass index (BMI) was calculated as weight (kg)/(height (m))^2^ and categorised into two: normal BMI (≥18.5 kg/m^2^) or low BMI (<18.5 kg/m^2^) [31].

### Ultrasound

Ultrasound examination of the urinary tracts of each child was performed by an experienced ultrasonographer using a portable convex sector scanner (SSD-500®; *Aloka*, Tokyo, Japan) according to the Niamey protocol [32] and bladder, ureter and kidney pathology was recorded. If any kidney and/ or ureter dilatation was observed the child was asked to empty the bladder and come back for re-examination.

### Statistical analyses

*S. haematobium* and STH egg counts were summed over the three samples, or for some just two samples, per child, and the amount of urine or faeces examined was used as an offset in the analyses of infection intensity. For graphical representation, egg counts were expressed as number of eggs per 10 ml urine (*S. haematobium*) or as eggs per g faeces (STH infections) and mean intensity and 95 % confidence limits were based on the average and 95 % confidence limits of log (x + 1) back transformed to the original scale. Since the recommended categories for intensity of infection [27, 28] produced highly uneven sample sizes for the different groups, we decided to cut intensity (from 1 to the maximum egg count) of each infection into three groups (low, medium and high) with approximately equal sample size. Zero counts (un-infected) constituted the base group against which the other three categories were compared. For *S. haematobium*, however, only 18 children were not infected and therefore this group was merged with the low intensity category.

Intensity of infection (as original counts) for each of the 4 infections was analyzed using negative binomial regression [33] in a generalized linear model with a log link function and with school, gender, age group and categories of infection intensity of the other three parasites as predictors. These predictors were all tested individually and later jointly in multi-variable models where initially all variables were entered and non-significant predictors (including non-significant levels of intensity of infection starting from the lowest category) were manually removed until only significant predictors were retained in the final model. Testing for over dispersion was based on the chi-square statistics [33]. Interactions between parasite intensity of infection (categories) was done pair-wise when main effects were significant without the interaction.

Similarly, prevalence of infection (not-infected/infected) for each infection separately was analyzed using logistic regression analysis [34] specified in a generalized linear model with a logit link function and otherwise following the same principle of modelling as above. Also for morbidity indicators (except body mass index), logistic regression analysis was done following the above guidelines including testing interactions between parasite species. BMI was analysed in a generalized linear model of the Gaussian family and with unit link function. For missing records of morbidity indicators, we always tested whether there was any bias between children sampled and those who were not in relation to the demographic variables and infections, but this was not the case. *P*-values less than 0.05 were taken to indicate significant effects.

## Results

### Study population and general infection levels

In total, 262 children with an average age of 9.7 years were recruited in the study. There were 128 boys and 134 girls and mean age in the two sexes was similar (9.9 vs. 9.6 years, respectively). A total of 96 children were aged between 5 and 9 years, and the other 166 children between 10 and 12 years. Of the 262 enrolled children, 242 provided three urine samples, each sample on a different day. The remaining 20 children provided two urine samples. All those who provided two samples only were positive for *S. haematobium* eggs. Two hundred and twenty-nine children provided three stool samples, each on a different day, whereas the other 33 provided two stool samples. Of those who provided two stool samples, 7 tested negative for STH eggs. All 262 children were included in the analyses.

**Fig. 1:**
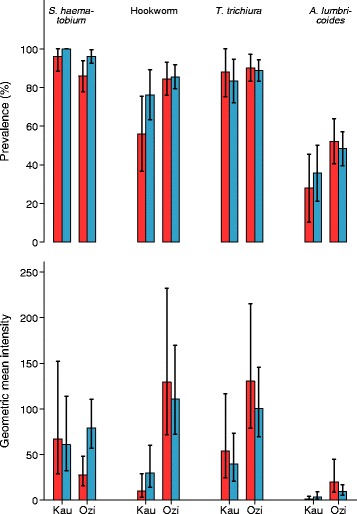
Prevalence (%) and geometric mean intensity of infection by school (Kau and Ozi) and age group. Red bars = 5–9 years and blue bars = 10–12 years. Geometric mean intensity given in no. of eggs per 10 ml urine (*Schistosoma haematobium*) or per g faeces (*Trichuris trichiura*, hookworm, and *Ascaris lumbricoides*). Error bars indicate 95 % confidence interval. Note that all 10–12 years old children were infected with *S. haematobium*; hence no error bar

The overall prevalence of *S. haematobium* was 94 %, with 38 % of the children having light (following WHO classification) and 56 % heavy infections; 81 % were infected with hookworm (75 % light and 6 % moderate to heavy), 88 % with *T. trichiura* (81 % light and 7 % moderate to heavy) and 45 % with *A. lumbricoides* (43 % light and 3 % moderate to heavy). The prevalence and intensity of *S. haematobium* and STH infections by school and age group are shown in Fig. 1.

### Analysis of infection prevalence and intensity

Prevalence and intensity of each of the four infections was first analysed separately using school, gender, age group and category of intensity of each of the other three infections as predictors (results shown in Table 1). Multivariable analyses were thereafter performed and the results of these are presented as follows.

**Table 1:** Association of *Schistosoma haematobium* and STH infections in the school children with various predictors. Coefficents are exponentiated regression coefficients from logistic regression (infection status) or from negative binomial regression (intensity of infection) as compared to the reference category, i.e., Kau school for school, girls for gender, 5–9 years for age class, non-infected plus light infection for *S. haematobium* and uninfected for the three other infections. Results from the multivariable analyses are presented in the text

		Infection status (odds ratio)				Intensity of infection (count ratio)			
	N	*S. haematobium*	Hookworm	*T. trichiura*	*A. lumbricoides*	*S. haematobium*	Hookworm	*T. trichiura*	*A. lumbricoides*
Ozi school	195	0.18	2.61**	1.45	2.02*	0.90	4.12***	3.65***	2.44
Boys	128	1.64	2.66**	1.37	1.35	1.22	1.97*	0.51***	1.53
Age 10–12 years	166	4.17*	1.47	0.80	0.97	1.34	0.90	0.50***	0.62
*S. haematobium*									
Medium: 35–231	82	---	0.98	1.41	1.21	---	1.67	0.46***	0.66
High: 232–1000	82	---	1.36	1.10	1.15	---	1.22	0.50**	0.97
Hookworm									
Low: 7–106	68	0.90	---	1.34	0.58	0.98	---	0.87	0.61
Medium: 113–453	73	0.47	---	11.21**	1.38	1.19	---	3.95***	7.58***
High: 460–3786	71	0.55	---	5.29**	2.59	1.14	---	3.35***	3.70*
*T. trichiura*									
Low:7–85	77	1.25	2.23	---	1.96	1.01	2.37*	---	0.82
Medium: 92–290	76	0.60	3.70*	---	3.09*	0.91	3.20**	---	2.19
High: 297–21231	78	0.26	4.84**	---	5.49***	1.00	4.96***	---	12.00***
*A. lumbricoides*									
Low: 7–43	38	0.38	0.74	2.35	---	1.12	1.96*	1.48	---
Medium: 50–297	41	0.55	1.55	2.55	---	1.15	1.85*	2.14**	---
High: 311–18789	40	0.53	2.39	7.87*	---	0.97	2.29**	5.67***	---

*p < 0.05; **p < 0.01; ***p < 0.001

For prevalence of *S. haematobium* infection, only age group remained significant, i.e., odds of infection in the 10–12 years age group was 4.17 (*p* < 0.05) of that in the 5–9 years age group. The final model for hookworm infection showed that school (OR = 3.10, *p* < 0.01), gender (3.13, *p* < 0.01) and *T. trichiura* infection (3.08, *p* < 0.01, respectively) were associated with higher odds of infection. The ORs for the three categories of *T. trichiura* infection intensity did not differ significantly and they were therefore pooled. Odds of *T. trichiura* infection was higher among those with medium and high hookworm infection intensity, i.e., odds of infection in children with medium intensity was 9.54 (p < 0.01) and in those with high intensity was 4.50 (*p* < 0.01) of that of children with no or low hookworm infection. For *A. lumbricoides* infection only high intensity of both hookworm (OR = 2.36, *p* < 0.01) and *T. trichiura* (2.18, *p* < 0.01) remained in the model. The interaction term between these two infections was not significant.

Intensity of *S. haematobium* infection did not show association with any of the predictors. Intensity of hookworm infection showed that school (CR = 4.17, *p* < 0.001) and gender (CR = 2.29, *p* < 0.001) were significant. Also in this model, medium and high intensity of *T. trichiura* infection were associated with higher intensity of hookworm infection, i.e., CR = 1.74 (*p* < 0.01) and 2.22 (*p* < 0.001), respectively. The dispersion statistics was 1.05, indicating a slight over-dispersion.

*T. trichiura* intensity was significantly associated with school (CR = 1.61, *p* < 0.001), gender (CR = 0.65, *p* < 0.01), and medium (CR = 2.37, <0.001) and high hookworm intensity of infection (CR = 3.49, <0.001). The *A. lumbricoides* egg count categories from negative to medium were combined and there was a significant interaction (*p* < 0.001) between this variable and *S. haematobium* egg count categories. The main effects of *S. haematobium* (i.e., the effect of *S. haematobium* intensity in children who had no to medium level of *A. lumbricoides* infection) was not significant, i.e., (CR = 0.79, *p* > 0.05) and (CR = 0.93, *p* > 0.05) for medium and high *S. haematobium* egg counts, respectively, while for children with high *A. lumbricoides* egg counts, *T. trichiura* counts were significantly lower when they also had medium (CR = 0.24, *p* <0.01) or high (CR = 0.15, *p* < 0.001) *S. haematobium* egg counts. These last two coefficients were not significantly different. The main effect of *A. lumbricoides* (i.e., in children who had no or light *S. haematobium* infection) was CR = 6.50 (*p* < 0.001). The model without the interaction was over dispersed (dispersion statistics =1.41), but although inclusion of the interaction improved the model (dispersion statistics =1.14), the model was somewhat over dispersed, so coefficients should be interpreted with care.

High *A. lumbricoides* intensity was significantly associated with gender (CR = 5.98, *p* < 0.001) and the *T. trichiura* intensity categories medium (CR = 4.74, *p* < 0.01) and high (CR = 39.53, *p* < 0.001). The dispersion statistics (1.20) indicated over-dispersion.

### Analysis of morbidity indicators

Associations between the various morbidity indicators and the various predictors individually are summarized in Table 2.

**Table 2:** Association of morbidity indicators in the school children with and various predictors. The coefficients reflect either mean difference (BMI) or odds ratios from logistic regression analysis between a given factor level and its corresponding baseline group. Results from the multivariable analyses are presented in the text

	BMI (*n* = 231)	Anaemia	Reported blood in urine	Macro- haematuria	Micro- haematuria	Eosinophilia	Bladder pathology	Upper urinary tract pathology	Total pathology
No. examined/no. affected	-	230/141	211/155	262/165	262/181	217/201	219/69	219/23	219/83
Ozi school	−0.34	1.70	1.53	0.65	0.85	1.24	1.15	0.64	0.82
Boys	0.11	1.03	0.91	2.14**	1.61	2.05	1.52	1.21	1.50
Age 10–12 years	0.84***	0.54*	1.87**	1.80**	1.87*	2.19	1.24	2.09	1.44
*S. haematobium*									
Medium: 35–231	0.12	1.87	4.67***	10.71***	16.13***	1.34	2.85**	0.58	2.17*
High: 232–1000	−0.48	2.08*	4.32***	279.82***	ne	2.34	4.40***	3.13*	4.40***
Hookworm									
Low:7–106	0.76*	1.17	0.29*	1.19	1.72	2.26	0.99	0.97	0.84
Medium: 113–453	0.23	1.60	0.40	1.34	1.65	3.44	1.85	0.90	1.63
High: 460–3786	−0.06	4.75***	0.32*	1.87	1.59	5.34*	1.35	1.53	1.42
*T. trichiura*									
Low:7–85	0.46	1.24	0.44	1.38	1.23	0.42	0.67	1.31	0.75
Medium: 92–290	0.68	1.80	1.01	0.80	0.53	0.61	0.78	0.16	0.70
High: 297–21231	0.51	1.63	0.50	0.75	0.70	0.56	0.62	2.53	0.88
*A. lumbricoides*									
Low: 7–43	−0.03	1.21	0.95	1.96	1.51	0.52	0.95	0.24	0.83
Medium: 50–297	−0.08	1.55	0.46	0.86	0.47*	1.15	1.41	0.78	1.22
High: 311–18789	−0.67*	1.21	0.78	0.82	0.83	1.05	0.99	1.79	1.45

*p < 0.05; **p < 0.01; ***p < 0.001

Weight and height measurements were available for 231 children. There was however no significant difference between the 231 and the remaining 31 children whose weight and height measures were not available in terms of infection prevalence and intensity. The great majority (95 %) of children were underweight (BMI < 11) and this created a problem for the statistical evaluation of the impact of infections. Therefore, we instead used the BMI scores directly. Higher BMI scores were associated with age and low hookworm infection intensity, while lower scores were associated with high intensity of *S. haematobium* and *A. lumbricoides* infection (Table 2). Age (b = 0.82, *p* < 0.001), low hookworm (b = 0.63, *p* < 0.01) and high intensity of *S. haematobium* infection (b = −0.57, *p* < 0.01) remained in multivariable model. The interaction between hookworm and *S. haematobium* infections was not significant.

Venous blood samples from 230 children were tested for haemoglobin levels. The overall prevalence of anaemia was 61.3 %. In multi-variable analysis, anaemia was associated with age group (OR = 0.47, *p* < 0.05), medium (OR = 2.25, *p* < 0.01) and high intensity (OR = 2.50, *p* < 0.01) of *S. haematobium* infection and high intensity of hookworm infection (OR = 3.74, *p* < 0.001).

A total of 211 children replied to whether they had seen blood in their urine in the past 3 months. Overall 73.5 % of the children reported to have seen blood in their urine. Blood in urine was associated with *S. haematobium* infection categories. This remained in the final model, i.e., medium (OR = 4.67, *p* < 0.001) and high (OR = 4.32, *p* < 0.001).

One hundred and sixty-five (63.0 %) of the 262 children had macro-haematuria, and were infected with *S. haematobium*. Of the 97 children with no macro re-treated -haematuria, only 16 (17 %) were not infected with *S. haematobium.* Higher odds of macro-haematuria were associated with gender and age group. Multi-variable analysis showed that odds of finding macro-haematuria was higher in boys (OR = 2.04, *p* < 0.05) than girls, and those having medium (OR = 10.6, *p* < 0.001) and high *S. haematobium* intensity of infection (OR = 276.06, *p* < 0.001) had higher odds of having macro-haematuria than those uninfected or with low intensity of *S. haematobium* infection.

The overall prevalence of micro-haematuria in the study population was 69 %. All children with micro-haematuria were infected with *S. haematobium* and none of the uninfected children had micro-haematuria whereas 26 % of infected children were negative for micro-haematuria. All children with high intensity of *S. haematobium* infection had micro-haematuria. This gave some problems in estimating coefficients in the logistic regression analysis. Therefore, for the multi-variable model medium and high intensity of *S. haematobium* were combined as one group. In the final model, the odds of children with medium and high intensity of *S. haematobium* having anemia was 46.13 (95 % CL: 19.77–107.63; p < 0.001) times that of children without or with light *S. haematobium* infection. In the same model children with medium/high intensity of *A. lumbricoides* infection had lower odds of anemia that children with no or light infection (OR = 0.27, 95 % CL: 0.11–0.64, *p* < 0.01).

White blood cell count was performed on blood samples from 217 children. The overall prevalence of eosinophilia (eosinophils ≥7 %) was 93 %. Odds of eosinophilia increased with hookworm infection intensity. None of the children had malaria parasites.

### Analysis of ultrasound detected morbidity

Of the 262 children examined for *S. haematobium* infection, the urinary tracts of 219 (84 %) were examined for morbidity using ultrasound (Table 2). Thirty-eight per cent of the children had different forms of urinary tract morbidity such as bladder wall thickness (31 %), bladder wall inner surface irregularity (30 %), bladder mass (7 %), pseudopolyps (2 %), kidney pyelon dilatation (11 %) and ureter dilatation (4 %). The former four morbidity types were combined into lower urinary tract pathology and the latter two types were combined into upper urinary tract pathology.

The odds of bladder pathology increased with intensity of *S. haematobium* infection (OR = 2.85, 95 % CL: 1.32–6.19, *p* < 0.01 and OR = 4.40, 95 % CL: *p* < 0.001 for medium and high intensity of infection, respectively). Multi-variable tests were not attempted due to the low number of cases with bladder pathology (Table 2).

Upper urinary tract pathology was associated significantly with high intensity of *S. haematobium* infection (Table 2) while total pathology did show significant association with *S. haematobium* infection and increased with intensity of *S. haematobium* infections.

Only 1 of the 16 children without *S. haematobium* infection had lower urinary tract pathology whereas 22 (11 %) of children with *S. haematobium* infection had upper urinary tract pathology and only one of those without the infection had upper urinary tract pathology.

## Discussion

The present study analysed infection and morbidity due to *S. haematobium* and STH in primary school children from two neighbouring villages in northern coastal Kenya. In agreement with estimates for the general area [9], the prevalence and intensity of *S. haematobium* infections among the children were high suggesting intense transmission. This could partly be due to extensive seasonal floods, under-developed transport infrastructure and low hygiene standards in the area. For example, less than 5 % of the households in the villages had latrines (data not shown) resulting in intense environmental contamination with helminth eggs. Children also extensively played in the nearby potentially snail-infested water bodies. Most infections with STH were of low intensity according to WHO classification [28]. Considering the low hygiene standards in the area, the reasons for this were not clear but could be due to harsh environmental conditions that did not favour survival and development of STH eggs and/or larval stages [14].

Only age group was found to be an important predictor for infection with *S. haematobium* among the children, with older children (10–12 years old) having higher odds than younger ones (5–9 years old). It is likely that older children had a higher tendency to get in contact with cercariae-infested water than younger children whose outdoor activities are more closely monitored and controlled by their parents. The finding that children in Ozi had higher odds of infection with hookworm than those in Kau suggests that different environmental factors influenced transmission in the two villages. It was observed that in Ozi, the soil was mainly sandy and covered with dense vegetation whereas in Kau the soil was mainly clay and sparsely covered with vegetation. These conditions are known to affect the survival and development of hookworm [35–38]. Boys in the two villages had a higher risk of infection with hookworm than girls suggesting that boys’ behaviour exposed them to infective hookworm larvae more than girls as also seen elsewhere [39]. For example, during field data collection in the present study it was observed that most of the boys, but not girls, could have been exposed when playing football with bared feet on moist soils. The association of *T. trichiura* and *A. lumbricoides* infections to school, gender and age could be explained by the same environmental and behavioural differences given above with boys and older children being more exposed to the infections than girls and young children, respectively, due to their tendency to spend more time outdoors.

No significant association was observed between *S. haematobium* and STH. This agrees with the findings of a study of children in Pemba Island where no correlation of infection intensity between *S. haematobium* and STH (*T. trichiura* and *A. lumbricoides*) was seen [40]. The reason could be that *S. haematobium* and STH occupy different habitats in the human host and therefore may have minimal immunological interaction. It is also possible that the different modes of transmission of *S. haematobium* and STH may account for the lack of interaction [14].

Children with higher intensity of *T. trichiura* had higher odds of infection with hookworm and vice versa. The eggs and larvae of the two parasites have more or less similar environmental requirements and the observation in the present study could therefore be due to epidemiological coincidence [14]. However, this does not explain why a similar phenomenon was not observed between the two STH (hookworm and *T. trichiura*) and *A. lumbricoides* which have similar epidemiology. Experimental studies have suggested that intestinal nematodes, including hookworms and *Trichuris* spp., can bias the host immune responses to favour their survival and avoid expulsion from host [41, 42]. This might favour heterologous intestinal nematode infections, thus resulting in synergistic interactions between hookworm and *T. trichiura*. In agreement with this, high intensities of hookworm and *T. trichiura* infections were also positively associated with *A. lumbricoides* infection status although the interaction terms were not statistically significant.

Prevalences of low BMI, anaemia, macro-haematuria, micro-haematuria and eosinophilia were remarkably high among the children. Similarly high prevalences of micro- and macro-haematuria were previously among schoolchildren in Kilifi, another area of coastal Kenya [18]. Haematuria was associated with *S. haematobium* infections in agreement with the suggestion that haematuria among schoolchildren in coastal Kenya is mainly due to infections with this parasite [43].

The prevalence of ultrasound detectable pathology among the children was high and together with a report from older schoolchildren from Kilifi [18] these findings indicate that urinary tract pathology among children in coastal Kenya is mainly due to infection with the parasite. Considering that ultrasound detects longstanding/chronic pathology [44, 45], the present study indicates that ultrasound-detectable pathology due to *S. haematobium* started to develop early in the lives of the children. Fortunately, late stage urinary tract morbidity such as hydronephrosis and hydroureter, which could potentially lead to renal failure, was not detected [46]. This suggests that treating children of this age group in the area to avert development of severe end-stage morbidity is important.

Infections with schistosomes and STH have been associated with poor nutritional status among children in endemic areas [47–50]. In the present study, higher BMI scores were associated with low hookworm intensity while lower scores were associated with high intensity of *S. haematobium* suggesting that infections with the two species played an important role in the nutritional status of the children. Although *A. lumbricoides* and *T. trichiura* have been associated with low BMI, a study in Ethiopia did not detect a significant association between the prevalence of STH and nutritional status [47, 48, 51]. In the present study there was no strong evidence to show that *A. lumbricoides* and *T. trichiura* contributed significantly to low BMI among the children probably because most of *A. lumbricoides* and *T. trichiura* infections were of low intensity [27, 28].

High intensities of *S. haematobium* and hookworm cause considerable blood loss and were associated with anaemia among the children [51–54]. Although *T. trichiura* and *A. lumbricoides* may cause anaemia [51, 53, 55] their intensities were low and thus their effects may have been obscured by the effects of *S. haematobium* and hookworms in the present study.

## Conclusion

The prevalence of *S. haematobium* and STH among primary school children in the study area was high with majority of *S. haematobium* infections being of high intensity whereas majority of STH infections were of low intensity. There was no evidence of association between *S. haematobium* and STH infections but hookworm and *T. trichiura* infections were positively associated. *S. haematobium* and hookworm were positively associated with anaemia and low BMI among the children. *S. haematobium* was moreover associated with early urinary tract morbidity, but late stage morbidity was not common. Increased coverage during mass administration of praziquantel and albendazole in the area is recommended to control morbidity due to these infections.
